# Diabetic Laryngitis

**Published:** 1900-06

**Authors:** 


### DIABETIC LARYNGITIS.

PATIENTS whose urine contains an appreciable amount of sugar, but whose general health has not yet suffered, will often consult the physician for a peculiar dryness of the throat and insufficiency of the voice after use. On examination the posterior pharyngeal wall is found to be dry, smooth, glistening and copper-colored, and the vocal cords have a peculiar shiny, glazed appearance. O. Leichtenstern (*Münch. med. Woch.*, April 17, 1900,) considers these signs as diagnostic of early diabetes. With proper dieting, the parts will return to the normal, showing that there merely is a hypersecretion without pathological change, although the latter may develop in the form of a sclerosis when the diabetes persists.—*Medical News*.

